# Supplementary material for: Sorting at embryonic boundaries requires high heterotypic interfacial tension
Source: Nat Commun. 2017 Jul 31;8:157. doi: 10.1038/s41467-017-00146-x (PMC5537356; doi:10.1038/s41467-017-00146-x)
Supplement: Supplementary file 2 — Supplementary Software 1 [file 41467_2017_146_MOESM2_ESM.zip › PottsModel/SrcPottsModel/doc/model/class-use/Constants.Model.html]

Uses of Class model.Constants.Model


JavaScript is disabled on your browser.


Skip navigation links


- Overview
- Package
- Class
- Use
- Tree
- Deprecated
- Index
- Help

- Prev
- Next

- Frames
- No Frames

- All Classes

## Uses of Class model.Constants.Model

- Packages that use Constants.Model

  | Package | Description |
  | --- | --- |
  |  |  |
  | --- | --- |
  | model |  |
- - ### Uses of Constants.Model in model

    Fields in model declared as Constants.Model

    | Modifier and Type | Field and Description |
    | --- | --- |
    |  |  |
    | --- | --- |
    | `Constants.Model` | Constants.ParameterManager.`model` |

    Methods in model that return Constants.Model

    | Modifier and Type | Method and Description |
    | --- | --- |
    |  |  |
    | --- | --- |
    | `static Constants.Model` | Constants.Model.`valueOf(java.lang.String name)` Returns the enum constant of this type with the specified name. |
    | `static Constants.Model[]` | Constants.Model.`values()` Returns an array containing the constants of this enum type, in the order they are declared. |

    Constructors in model with parameters of type Constants.Model

    Constructor and Description

    `EnergyManager(Constants c,
    Constants.Model m,
    PottsEngine.StateVariables s)`

Skip navigation links


- Overview
- Package
- Class
- Use
- Tree
- Deprecated
- Index
- Help

- Prev
- Next

- Frames
- No Frames

- All Classes
